# Construction and validation of a metabolism-associated gene signature for predicting the prognosis, immune landscape, and drug sensitivity in bladder cancer

**DOI:** 10.1186/s12920-023-01678-6

**Published:** 2023-10-26

**Authors:** Chong Shen, Yuxin Bi, Wang Chai, Zhe Zhang, Shaobo Yang, Yuejiao Liu, Zhouliang Wu, Fei Peng, Zhenqian Fan, Hailong Hu

**Affiliations:** 1 Department of Urology, The Second Hospital of Tianjin Medical University, 23 Pingjiang Road, Jianshan Street, Hexi, Tianjin, 300211 People’s Republic of China; 2 Tianjin Key Laboratory of Urology, Tianjin Institute of Urology, Tianjin, 300211 China; 3 Department of Pharmacy, Zhu Xianyi Memorial Hospital of Tianjin Medical University, Tianjin, China; 4 Department of Critical Care Medicine, the Peoples Hospital of Yuxi City, Yunnan, China; 5 Department of Endocrinology, The Second Hospital of Tianjin Medical University, 23 Pingjiang Road, Jianshan Street, Hexi, Tianjin, 300211 People’s Republic of China

**Keywords:** Bladder cancer, Metabolism, Prognostic model, Tumor microenvironment, Immune therapy

## Abstract

Tumor Metabolism is strongly correlated with prognosis. Nevertheless, the prognostic and therapeutic value of metabolic-associated genes in BCa patients has not been fully elucidated. First, in this study, metabolism-related differential expressed genes DEGs with prognostic value in BCa were determined. Through the consensus clustering algorithm, we identified two molecular clusters with significantly different clinicopathological features and survival prognosis. Next, a novel metabolism-related prognostic model was established. Its reliable predictive performance in BCa was verified by multiple external datasets. Multivariate Cox analysis exhibited that risk score were independent prognostic factors. Interestingly, GSEA enrichment analysis of GO, KEGG, and Hallmark gene sets showed that the biological processes and pathways associated with ECM and collagen binding in the high-risk group were significantly enriched. Notely, the model was also significantly correlated with drug sensitivity, immune cell infiltration, and immunotherapy efficacy prediction by the wilcox rank test and chi-square test. Based on the 7 immune infiltration algorithm, we found that Neutrophils, Myeloid dendritic cells, M2 macrophages, Cancer-associated fibroblasts, etc., were more concentrated in the high-risk group. Additionally, in the IMvigor210, GSE111636, GSE176307, or our Truce01 (registration number NCT04730219) cohorts, the expression levels of multiple model genes were significantly correlated with objective responses to anti-PD-1/anti-PD-L1 immunotherapy. Finally, the expression of interested model genes were verified in 10 pairs of BCa tissues and para-carcinoma tissues by the HPA and real-time fluorescent quantitative PCR. Altogether, the signature established and validated by us has high predictive power for the prognosis, immunotherapy responsiveness, and chemotherapy sensitivity of BCa.

**Supplementary Information:**

The online version contains supplementary material available at 10.1186/s12920-023-01678-6.

## Introduction

The prevalence and mortality rates of bladder cancer (BCa) are high. It is estimated that currently > 1.6 million individuals have BCa worldwide, with approximately 550,000 new cases annually [1]. It is anticipated that the incidence of BCa will be extremely rapid with increasing life expectancy and the increase of risk factors such as smoking in the developing countries. Despite research in recent years has made remarkable progress in our understanding of cancer, the burden of it is increasing.

Tumor cells reprogram the metabolic pathway to meet the bioenergetic, biosynthetic, and redox demands of malignant cells [2]. The Warburg effect shows that a unique metabolic phenotype was identified for cancer cells, featuring that compared to normal tissues, they are characterized by a high rate of glycolytic metabolism. Recent evidence suggests that metabolic deregulation may not only be a characteristic feature of human cancers but also a potential underlying factor [3]. The model of metabolic genes have been reported in pancreatic cancer [4], prostate cancer [5], hepatocellular carcinoma [6], colon cancer [7], and other cancer. However, the effects of metabolic changes on BCa progression are unknown. Recently, the new biomarkers used in drug screen of immunotherapy were introduced into clinical practice, which has shown favorable prospects in treating to some degree. However, the reaction to immunotherapy remains poorly elucidated.

In the current study, we first analyzed metabolism-associated prognostic DEG genes in bladder cancer, then constructed and validated a new signature across multiple datasets, and finally explored the prognostic value of the model and its relationship with the clinicopathological features, immune microenvironment, and the therapeutic effect of chemotherapy agent and immunotherapy of patients with BCa. We also confirmed the mRNA and protein expression of interested model-related genes from the Human Protein Atlas (HPA) and 10 paired BCa tissues collected by us. The most valuable aspect of the research is that our developed model provides more information for the selection of clinical treatment options and prognostic prediction.

## Materials and methods

### Clinical specimens

From September 2021 to October 2022, a total of 10 cases of BCa and its matched adjacent non-tumor tissues were collected in the Second Affiliated Hospital of Tianjin Medical University (China). The final diagnosis of each patient was confirmed by histopathology. In this study, the written informed consent of each patient or their guardian was obtained following the guidelines approved by the Medical Ethics Committee of the Second Hospital of Tianjin Medical University.

### Data collection and processing

The datasets used to construct or validate metabolism-relevant gene signatures for BCa come from five different platforms: Cancer Genome Atlas (TCGA, https://portal.gdc.cancer.gov/repository) and Gene Expression Omnibus (GEO, https://www.ncbi.nlm.nih.gov/geo/). According to the TCGA database, 406 BCa samples were downloaded, including mRNA expression profile data and clinical information. Gene annotation is made possible through the Ensemble database. The microarray expression data of GEO datasets such as GSE13507, GSE32894, GSE48075, GSE31684, GSE111636, and GSE176307 were quantified and positiveized. IMvigor210 dataset, a cohort of 348 MIBC patients treated with atezolizumab (PDL1 inhibitor), is also used to verify metabolism-related gene signatures.

Notably, in our single-arm phase 2 trial (term_id, TRUCE-01; registration number, NCT04730219), tislelizumab (200 mg) combined with low-dose nab-paclitaxel (200 mg) also preliminary confirmed clinical benefits and safety in the therapy of muscle-invasive urothelial bladder carcinoma patients. Therein, tislelizumab is a novel humanized monoclonal antibody programmed death receptor-1 (PD-1) inhibitor and shows a predictable and manageable safety/tolerability profile in patients with PD-L1^+^ UC.

### Extraction of metabolism-related prognostic genes, identification of BCa subclasses, and its association with infiltration of immune cells

We used cancer versus normal differential analysis to find metabolism-associated genes differentially expressed (DEGs) in tumor tissue and tumor surrounding tissues, and set |log(fold change, FC)|> 1, false-discovery rate (FDR) < 0.05 as the threshold to construct the heat map. Then, we performed a univariate Cox regression analysis on these DEGs.

NMF was used to identify subtypes of molecules and obtained the molecular subtypes C1, and C2 for BCa, and the log-rank test was applied. Kaplan–Meier survival analysis was used to compare the overall survival curves (OS), progression-free survival (PFS), disease-free survival (DFS), and disease-specific survival (DSS) between C1 and C2.

To analyze the immune status of each sample, Cell-type Identification By Estimating Relative Subsets Of RNA Transcripts (CIBERSORT) was used to calculate the relative proportions of 22 immune cells in BCa patients, which include seven T cell types, naïve and memory B cells, plasma cells, NK cells, and myeloid subsets. CIBERSORT was applied to convert mRNA data into the proportions of infiltrating non-tumor cells in the tumor microenvironment using standard annotation files to organize gene expression characteristics. Afterward, correlation analysis was conducted between the above molecular typing and immune cell infiltration estimated by CIBERSORT.

### Establishment and validation of the metabolism-related prognostic prediction model

Then, the TCGA BCa cohort was randomly divided into a training set (*n* = 285) and a testing set (*n* = 121) at a ratio of 7:3. Chi-squared tests were employed to compare baseline characteristics between the training set and external validation set. Ten prognostic DEGs were screened out using univariate Cox regression and the Least Absolute Shrinkage and Selection Operator (LASSO) regression analysis. Genes with *P* < 0.05 have prognostic significance. Then we constructed a risk prediction model with the following formula: risk score = 0.267*PLOD1 + 0.167*SERPINB7 + 0.160*TSPAN7 + 0.291*HSD17B1 + 0.132*PYCR1 + 0.266*PDGFRA + 0.107*EGR1 + 0.537*AHCY + 0.121*ATP6V1B1 + 0.202*SCD. Next, we divided BCa patients into high-risk and low-risk groups according to the median risk score. Across TCGA-training set, internal TCGA-testing and multiple external GEO validation (including GSE13507, GSE32894, GSE48075, GSE31684, and IMvigor210), Kaplan–Meier (KM) survival and time-dependent receiver operational feature (ROC) curves were plotted through “survival”, and “timeROC” R packages to estimate the discrimination of the metabolism-related 10-gene signature. Then, calibration curves were also generated to assess the stability of the models. The metabolism-related gene model was established by referring to previous studies [8]. Subsequently, the some further analytic strategy on this model, such as the pathway enrichment analysis, and immune infiltration-related analysis were also similar to a previously published researchs [8, 9].

### Correlations between the model and clinical features

Kaplan–Meier survival analyses for BCa patients of the high- and low-risk groups were carried out in the different clinical subgroups. In addition, univariate and multivariate Cox regression analyses confirmed that risk score was an independent prognostic predictor of OS. A combined nomogram model was further constructed to predict the 1-, 3- and 5-year OS rates of BCa patients. This nomogram included the risk scores and common clinical information such as grade, gender, age, and stage.

### Predictive combined nomogram model construction

Using the “rms” package of the R software, a calibration chart was generated to verify the predictive power of the nomogram. Nomogram accuracy was evaluated using ROC curve analysis. A decision curve analysis (DCA) was then performed to assess the clinical usefulness of the predictive model. Furthermore, we compared the predictive power of our risk model with the previous two human models, including the tumor immune dysfunction and exclusion (TIDE) TIDE and the tumor inflammation signature (TIS).

### GO, KEGG, and Hallmark genesets enrichment analyses via GSVA method based on TCGA, GSE13507, and GSEA32894

We explored the GO, KEGG, and Hallmark enrichment differences between the high- and low-risk groups by GSVA algorithm based on the TCGA dataset [10–13]. Of them, GO analysis includes molecular function (MF), cellular component (CC), and biological process (BP). Pathways with *p* < 0.05 were considered significantly enriched. Additionally, two additional different datasets (i.e., GSE13507 and GSEA32894) were interrogated to identify enriched functional and pathway terms.

### Correlation analyses between the metabolism-related gene model and the tumor microenvironment and expression of immune checkpoint genes

We also used ESTIMATE’s algorithm to evaluate immune and stromal fractions to reflect the ratio of immune cells to stromal cells. Pearson’s correlation was used to analyze the correlation between the risk score of the signature and above immune or stromal scores. We evaluated also the expression differences of the multiple immune checkpoint genes, such as PD-1, PD-L1, CTLA4, etc., between high- and low-risk groups. Immune cell infiltration of tumor samples was identified using TIMER 2.0 (cistrome.shinyapps.io/timer/) via the MCPCOUNTER, CIBERSORT, QUANTISEQ, TIMER, CIBERSORT-ABS, EPIC, and XCELL algorithms. Wilcox rank sum test was used to analyze the difference in the level of each immune cell between the two risk groups. Spearman rank correlation analysis was applied to analyze the correlation between risk scores and immune infiltrating cell scores.

### Association analyses of the model with additional immunological characterization

The tumor immune dysfunction and exclusion (TIDE) algorithm [14] based on the simulation of tumor immune evasion mechanism was used to analyze the expression level of relatively more important immunological checkpoint genes (including PD1, PDL1, VISTA, TIGIT, and CTLA4, etc.), which have been identified and developed for the treatment of cancer. Moreover, the TIDE was employed to analyze multiple signatures to estimate tumor immune evasion, such as exclusion, or CAF signatures. The immunotherapy response of cancer patients was predicted by the above multiple TIDE scores via the Wilcox test. PD1 and CTLA4 immune therapy agents can be predicted using the immunophenoscore (IPS). In addition, we also analyzed associations of the model with tumor mutation burden (TMB) and immunophenoscore (IPS), which are all superior predictors of the response to immune checkpoint blockades (anti-PD1, anti-CTLA4, etc.). Hence, we could predict the immunotherapy response of each BCa patient using our model.

### Screening potential chemotherapy agents based on our prediction model

First, we downloaded the drug sensitivity information from the CellMiner database to explore the connection between modeled gene expression levels and drug susceptibility. Drugs approved by the FDA or clinical trials were selected for analysis. Next, the “pRRophetic” package was applied to analyze and compare the half-maximal inhibitory concentration (IC50) between two risk score groups to Doxorubicin, Docetaxel, Paclitaxel, and Vinblastine.

### Correlation analysis of ten modeled genes with clinical immunotherapy efficacy across four independent cohorts

We drew a boxplot of model-related gene expression levels in the responder vs. non-responder groups based on Imvigor210, GSE111636, GSE176307, and Truce01 datasets. Notely, we also conducted a comparison of model-related gene expression differences after vs. before the treatment using a paired Wilcoxon test based on our Truce01 sequencing data. The statistical difference was set at *p* ≤ 0.05.

### The expression validation of the model genes by Human Protein Atlas (HPA) and quantitative real-time PCR (qRT-PCR)

The protein expression levels of the ten model genes were verified through the HPA databases (HPA: https://www.proteinatlas.org/), and the consistency between the transcriptome described above and proteome levels was observed.

RNA extraction, quantitative reverse-transcription PCR, and human protein atlas (HPA) analysis. Total RNA was extracted from 10 paired BCa tumors and adjacent tissues via E.Z.N.A.TM Hp total RNA Kit (OMEGA); and was reversed into cDNA using RevertAid First Strand cDNA Synthesis Kit (Thermo Fisher Scientific, Rockford, IL, USA). Quantitative reverse-transcription PCR (qRT-PCR) was used to determine the relative expression of EGR1, PLOD1, and PYCR1 mRNAs in 10 BCa tissues compared to matched paracancerous tissues. The EGR1 primer sequences were: forward, 5’-GGTCAGTGGCCTAGTGAGC-3’; reverse, 5’-GTGCCGCTGAGTAAATGGGA-3’. PLOD1 primer: forward, 5’-AGACCAAGTATCCGGTGGTGT-3’; reverse, 5’- CTTGAGCACGACCTCATCCAA-3’. PYCR1 primer: forward, 5’-CTTCACAGCAGCAGGCGTC-3’; reverse, 5’-TCTCCTTGTTGTGGGGTGTC-3’. GAPDH was used as a control gene: forward primer, 5’-CGGAGTCAACGGATTTGGTC-3’; reverse primer, 5’-TTCCCGTTCTCAGCCTTGAC-3’. The final results were analyzed using the 2^−ΔΔCT^ method.

### Statistical analysis

R software version 4.1.0 was used for all statistical analyses. Using Wilcox’s test, we compared the two groups’ variables. Chi-square tests have been used to examine the association of risk groups with clinicopathological features. The survival data were assessed using the Kaplan–Meier curve. The ROC analysis was conducted using the R package time. An univariate and multivariate Cox regression analysis was conducted to evaluate independent prognostic factors. *P* ≤ 0.05 was considered to be statistically significant. Furthermore, *p*-value summaries were as follows: *P* > 0.05 (ns); *P* ≤ 0.05 (*); *P* < 0.01 (**); *P* < 0.001 (***); *P* < 0.0001 (****).

## Result

### Differentially expressed metabolism-related prognostic genes in BCa

**Fig. 1:**
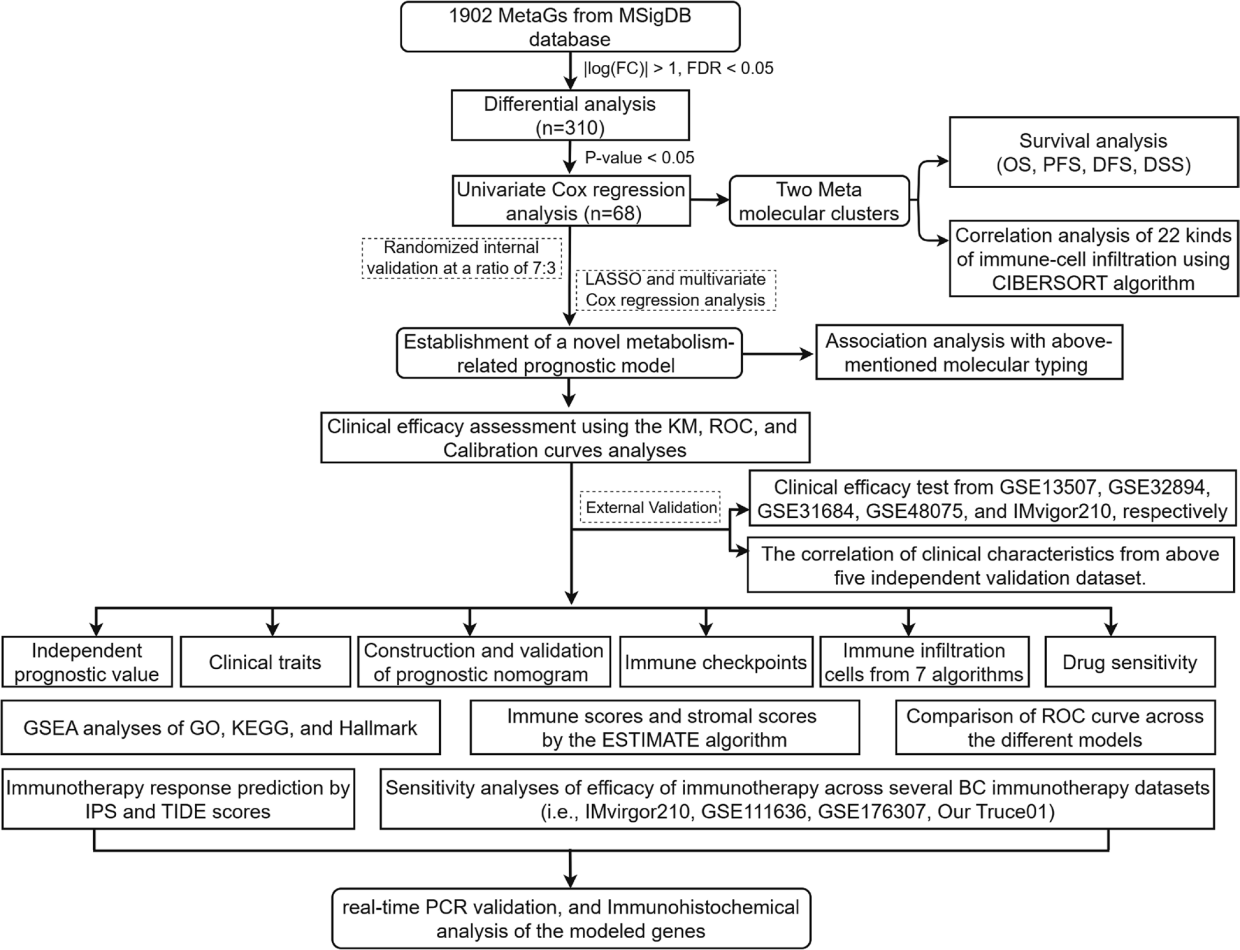
Flowchart of this study. MetaGs, Metabolism-Associated Genes; MsigDB, The Molecular Signatures Database; OS, Overall survival curves; PFS, Progression free survival; DFS, Disease freesurvival; DSS, Disease specific survival; LASSO, Least absolute shrinkage and selection operator; KM, Kaplan–Meier method; ROC, Receiver operating characteristic; GSEA, Gene set enrichment analysis; GO, Gene ontology; KEGG, Kyoto encyclopedia of genes and genomes; TIDE: The tumor immune dysfunction and exclusion; IPS: Immunophenoscore

**Fig. 2:**
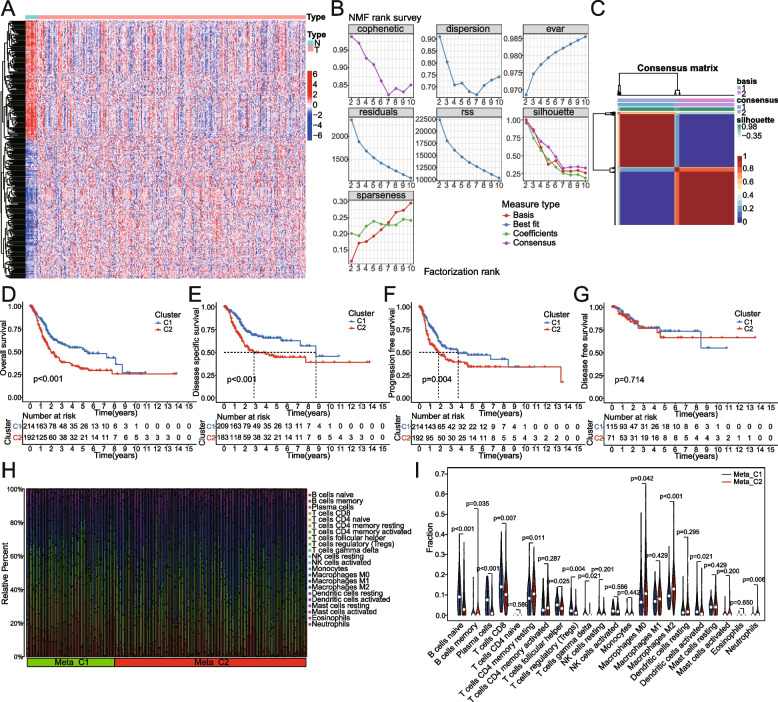
Identification of metabolism-related molecular subtyping, and its correlation with immune cells infiltrating based on TCGA database. **A** A heat map of metabolism-related differentially expressed genes in bladder cancer versus pericarcinoma tissues. **B-C** The NMF rank survey and consensus matrix heatmap were presented. Rank = 2 is the optimal number of clusters. **D-G** Differences of two subclasses (C1 and C2) were determined by log-rank test in OS, DSS, PFS, and DFS. **H** The relative percentage of 22 immune cells was estimated by the CIBERSORT algorithm for each BCa sample.** I** Comparison of the difference in the number of each immune-cell infiltration between the two clusters by Wilcoxon tests. N, normal sample; T, tumor sample

This study's general step-by-step process is illustrated in Fig. 1. We collected 1314 metabolism-associated genes from TCGA, and 406 metabolism-associated genes with differential expression were identified using the R project “Limma” (| logFC |> 1, FDR < 0.05) **(**Fig. 2A). Subsequently, a total of 68 OS-related genes were detected via the univariate Cox regression (*P* < 0.05).

### Molecular typing based on metabolism-related prognostic DEGs

BCa patients were clustered into two distinct subtypes using the ‘NMF’ algorithm, which is an effective method for reducing dimension in cancer subtype identification (Fig. 2C). The optimal number of clusters was determined as k = 2 after the NMF rank survey (Fig. 2B; Supplementary Figure S1). The results showed that patients with the C1 subtype had a more favorable prognosis of OS, DSS, and PFS than those with the C2 subtype (Fig. 2D-G). To explore the differences in immune cell infiltration between the two subtypes, we also evaluated 22 kinds of immune cells by the CIBERSORT algorithm. There were significant differences in infiltration levels between the two groups for many types of immune cells. For example, cluster 1 presented higher infiltration of naive B cells, plasma cells, CD8 + T cells, follicular helper T cells, Tregs, and activated dendritic cells, and cluster 2 exhibited greater infiltration of memory B cells, CD4 + memory resting T cells, M0 Macrophages, M2 Macrophages, resting dendritic cells and neutrophils (Fig. 2H, I).

### Construction and validation of a prognostic prediction model of metabolism-related genes

**Fig. 3:**
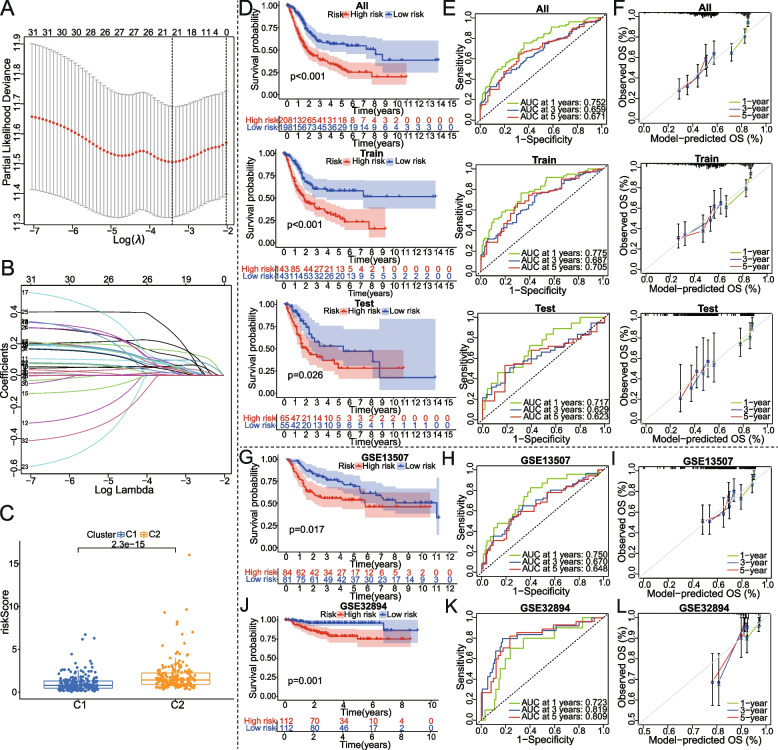
Construction and validation of a metabolism-associated prognostic model. **A**, **B** The tenfold cross-validation for variable selection with minimal lambda value in the LASSO regression. **C** Differences in risk score between subtype C1 and C2. **D-F** KM survival analysis, time-dependent ROC curves, and calibration curves for OS were all performed in the high- and low-risk groups based on TCGA_entire, TCGA_training, and TCGA_testing datasets. **G-L** Similarly, ROC, KM, and calibration curves of two additional datasets are shown

We randomly divided the TCGA BCa cohort into training and internal testing sets at a ratio of 7:3 (*n* = 286, and *n* = 120, respectively). Based on the chi-square test, there were no significant differences between the training and testing sets (Table 1). Then, the prognostic model of ten metabolism-related genes (PLOD1, SERPINB7, TSPAN7, HSD17B1, PYCR1, PDGFRA, EGR1, AHCY, ATP6V1B1, and SCD) was successfully constructed using the lasso and multivariate cox regression based on TCGA dataset (Fig. 3A, B). It can be seen that the C2 subtype had a higher risk score than the C1 subtype (Fig. 3C). BCa patients with high risk survived significantly shorter times than low-risk patients, regardless of whether in a training group, a test group, or an entire group (Fig. 3D). The performance of the KM survival curves was further validated in the external validation cohorts (GSE13507, and GSE32894) (Fig. 3G, J). Through the use of ROC curves, the model’s performance was evaluated. The area under the ROC curve (AUCs) of 1-, 3-, and 5-year were 0.752, 0.659, and 0.671, respectively, in the TCGA-entire set; 0.775, 0.687, and 0.705, respectively, in the TCGA-training set; 0.717, 0.629, and 0.623, respectively, in the TCGA-testing set; 0.750,0.670 and 0.648, respectively, in the GSE13507 validation set; and 0.723, 0.819, and 0.809, respectively, in the GSE32894 validation set (Fig. 3E, H, K). Additionally, the calibration curves of the prediction model were close to the standard curves in TCGA-entire, TCGA-training, TCGA-testing, and two external geo-validation cohorts, separately (Fig. 3F, I, L).

**Table 1:** Chi-square tests were used to compare frequencies of clinicopathological variables between the train and test groups

Clinical traits	Type	Total (*n* = 406)	Train (*n* = 286)	Test (*n* = 120)	*P* value
Age	< = 65	160(39.41%)	114(39.86%)	46(38.33%)	0.8603
	> 65	246(60.59%)	172(60.14%)	74(61.67%)	
Gender	Female	107(26.35%)	74(25.87%)	33(27.5%)	0.8291
	Male	299(73.65%)	212(74.13%)	87(72.5%)	
Grade	High Grade	383(94.33%)	269(94.06%)	114(95%)	0.8384
	Low Grade	20(4.93%)	15(5.24%)	5(4.17%)	
	unknown	3(0.74%)	2(0.7%)	1(0.83%)	
Stage	Stage I-II	131(32.27%)	97(33.92%)	34(28.33%)	0.201
	Stage III	140(34.48%)	91(31.82%)	49(40.83%)	
	Stage IV	133(32.76%)	97(33.92%)	36(30%)	
	unknown	2(0.49%)	1(0.35%)	1(0.83%)	
T	T1-2	122(30.05%)	89(31.12%)	33(27.5%)	0.1039
	T3	193(47.54%)	127(44.41%)	66(55%)	
	T4	58(14.29%)	46(16.08%)	12(10%)	
	unknown	33(8.13%)	24(8.39%)	9(7.5%)	
M	M0	195(48.03%)	143(50%)	52(43.33%)	0.7226
	M1	11(2.71%)	7(2.45%)	4(3.33%)	
	unknow	200(49.26%)	136(47.55%)	64(53.33%)	
N	N0	236(58.13%)	164(57.34%)	72(60%)	0.4069
	N1-3	128(31.53%)	95(33.22%)	33(27.5%)	
	unknow	42(10.34%)	27(9.44%)	15(12.5%)	

*T* T-stage, *N* stage of lymph node metastasis, *M* metastatic stage

### Correlations of the prognostic model established by 10 metabolism-related genes with clinical characteristics

**Fig. 4:**
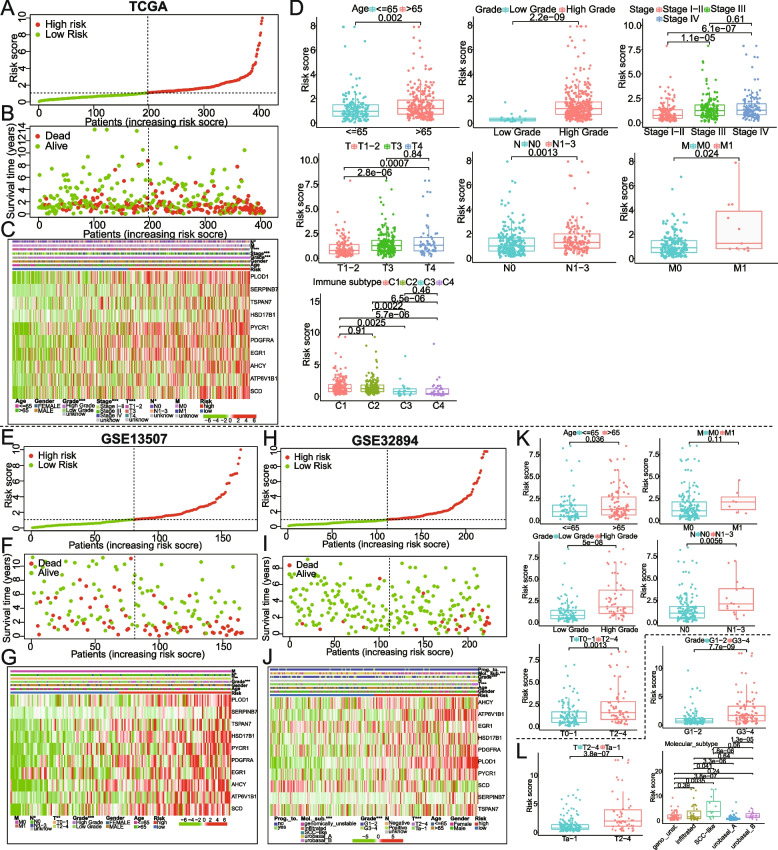
**A-C** Based on the TCGA_BCa cohort, it was displayed from top to bottom including risk score curves, survival status, and model gene expression heatmap with the correlation between risk group and clinical features. The horizontal axis of these graphs is sorted by risk score value. **D** The scatter diagram demonstrated that risk scores were statistically significant in different groups by age, tumor grade, M_stage, T_stages, or N_stages. **E-L** The analyses mentioned above were also implemented in the validation sets GSE13507 and GSE32894

Subgroup analysis results showed that the model had good predictive efficacy in different age, stages, gender, or stage_T groups (*P* < 0.05) (Supplementary Figure S2). Our univariate and multivariate Cox regression analysis exhibited that the risk score of the model can serve as an independent prognostic factor (*p* < 0.0001; Table 2). According to the TCGA cohort, the risk scores for each BCa sample were ranked from low to high, with a higher risk score indicating a higher risk of death (Fig. 4A, B). Expression levels of ten model genes showed a trend of increase with the risk score from low to high (Fig. 4C). To investigate the relationship between the gene signature and clinical features, we compared the risk scores between different clinical features using chi-square or Wilcoxon nonparametric tests across the TCGA and GEO datasets. As illustrated in Fig. 4D, patients older than 65 years have a higher risk score than patients younger than 65 years of age. Moreover, patients with high grades exhibited higher risk scores than patients with low grades. BCa patients with M1, N1-3, or T3-4 displayed a higher risk score than M0, N0, or T1-2, respectively. Furthermore, immunotyping of wound healing (C1) and interferon-gamma dominant (C2) may happen in patients with the high-risk score, while the inflammatory (C3), and lymphocyte depleted (C4) are more likely to appear in those with low-risk score (*p* < 0.05, Fig. 4D).

**Table 2:** Univariate and multivariate Cox regression analysis of exosome-associated prognostic model (risk score) and clinical characteristics in patients with BCa

Variables	Univariate analysis (OS)		Multivariate analysis (OS)	
	**HR (95% CI)**	***p*-value**	**HR (95% CI)**	***p*-value**
Age (Conti. vari.)	1.034 (1.018–1.051)	2.11E-05	1.029 (1.013–1.045)	0.0003
Gender (Male/Female)	0.894 (0.644–1.241)	0.504		
Histo. gr. (High/Low)	2.881 (0.713–11.647)	0.138		
Stage (III-IV/II/I)	1.743 (1.436–2.115)	1.95E-08	1.578 (1.296–1.923)	5.84E-06
Risk score (Conti. Vari.)	1.338 (1.260–1.422)	2.58E-21	1.298(1.216–1.385)	4.59E-15

*OS* overall survival, *HR* hazard ratio, *CI* confidence interval, *Conti. Vari.* continuity variables, *Histo. gr.* histologic grade

The risk scores, survival status, and heatmap of model gene expression were also performed using the GSE13507 and GSE32894 datasets, and the results were almost consistent with the above analysis findings of TCGA (Fig. 4E-J). The correlation results between risk score and clinicopathological parameters in GSE13507 (Fig. 4K) or GSE32894 datasets (Fig. 4L) were nearly consistent with the above analysis from the TCGA datasets. Additionally, we also compared the risk score among Sjodahl et al.’s five molecular classifications and found that the SCC-like group had a higher risk score (Fig. 4L).

**Fig. 5:**
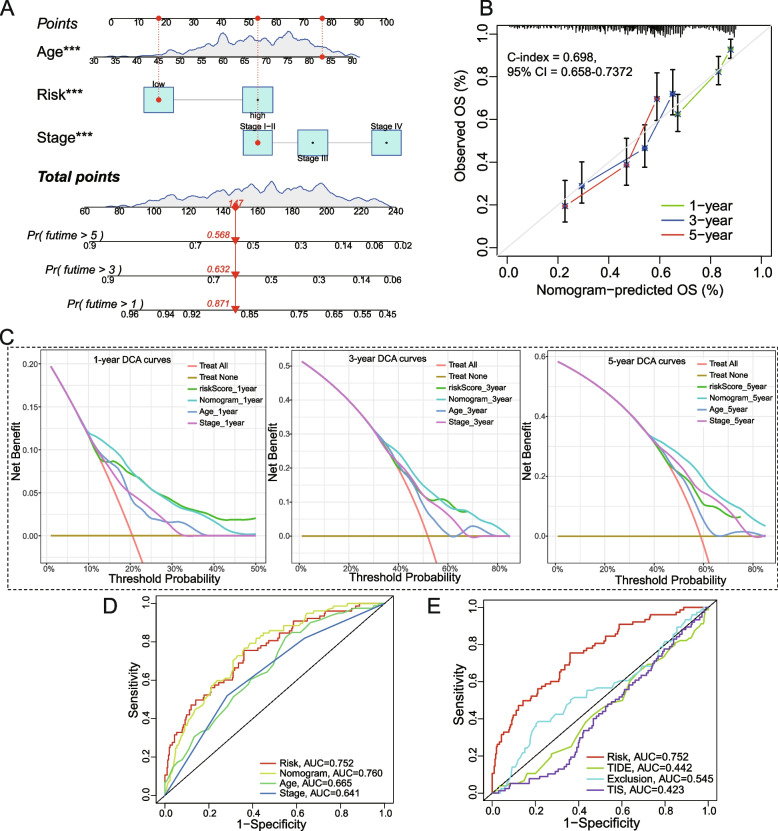
The nomogram construction and validation are based on the TCGA database. **A** The nomogram of risk scores and clinical characteristics with independent prognostic prognosis (i.e., age and stage) were established to forecast the probability of 1-, 3-, and 5-year OS. **B** The 1-, 3-, or 5-year calibration curves for nomogram model. **C** The clinical net benefit decision curve of the nomogram for the 1-, 3-, and 5-year OS. **D** The 1-year OS ROC curves of risk score, multiple clinical features, and nomogram. **E** Comparison of ROC curves among TIS, TIDE, Exclusion, and our risk model. C-index, the concordance index; CI, confidence interval; DCA, decision curve analysis; AUC, area under the curve; TIDE, the tumor immune dysfunction and exclusion; TIS, the tumor inflammation signature. T. ***P < 0.001

Based on risk group, age and stage, we established a combined nomogram model for predicting the 1-, 3-, and 5-year OS incidences of BCa patients (Fig. 5A). We also utilized calibration plots to assess and confirm the concordance between the nomogram-predicted and actual 1-, 3-, and 5-year OS (Fig. 5B). The concordance index (C-index) for the nomogram was 0.698 (95% CI: 0.658–0.7372). Furthermore, Decision Curve Analysis (DCA) of 1-, 3-, and 5-year OS for the model further demonstrated our expectations. It was found that compared to a single clinical factor, the model and combined nomogram showed the highest clinical net benefit (Fig. 5C). Multivariate time-dependent ROC curve according to age, gender, grade, stage, nomogram, and risk score, is presented in Fig. 5D. Notely, compared to TIDE, Exclusion, and TIS prediction models from previous studies, the signature constructed by us presented a better sensitivity (Fig. 5E).

### Function enrichment analysis of the model

**Fig. 6:**
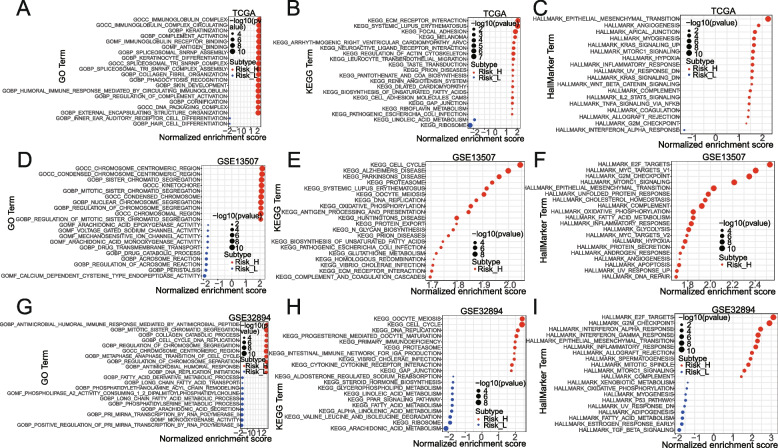
GSEA enrichment analysis for GO, KEGG, and Hallmark genesets in different risk groups based on the TCGA (**A-C**), GSE13507 (**D-F**), and GSE32894 (**G-I**). Risk_H, Risk_high; Risk_L, Risk_low

To explore the underlying molecular mechanism of the metabolism-related gene model, we next conducted GSEA for GO, KEGG, and HALLMARK gene sets, and further identified differences in biological function and pathways between the high- and low-risk groups based on the TCGA, GSE13507, or GSE32894 cohort (Fig. 6, Supplementary Table S1). For GO analysis results, ‘immunoglobulin complex circulation’, ‘complement activation’, ‘immunoglobulin receptor binding’, ‘complement activation’, and ‘spliceosomal snRNP assembly’ were enriched in the high-risk group (Fig. 6A, D, G). KEGG pathways enrichment analysis revealed that ‘ECM receptor interaction’, ‘systemic lupus erythematosus’, ‘focal adhesion’, ‘melanoma’, ‘arrhythmogenic right ventricular cardiomyopathy arvc’, ‘neuroactive ligand-receptor interaction’, ‘regulation of actin cytoskeleton’, ‘leukocyte transendothelial migration’ and ‘taste transduction’ were particularly significant in the high-risk group (Fig. 6B, E, H). Among them, the most significantly enriched HALLMARK terms (‘epithelial-mesenchymal transition’, ‘angiogenesis’, ‘apical junction’, ‘myogenesis’, ‘hypoxia’, ‘inflammatory response’, etc.) is shown in Fig. 6C, F, I.

### The external validation of the metabolism-associated gene signature based on additional three cohorts

**Fig. 7:**
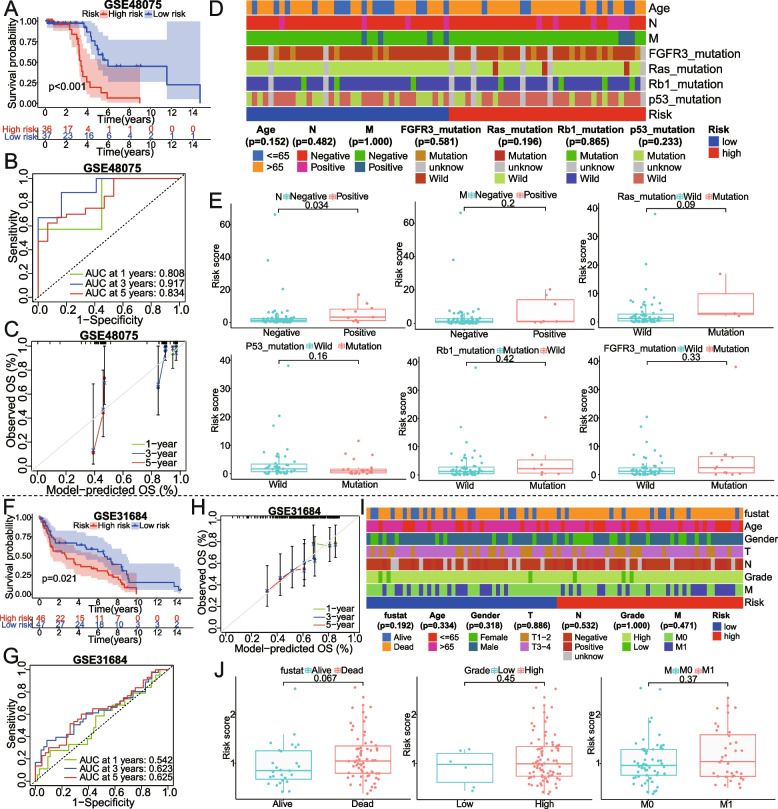
The KM, ROC, calibration curves, and the correlation of clinical traits for the model were also analyzed in two external verification sets, **A-E** GSE48075 and **F-J** GSE31684. AUC, area under the curve; OS, overall survival; T, T-stage; N, stage of lymph node metastasis; M, metastatic stage; fustat, survival status

**Fig. 8:**
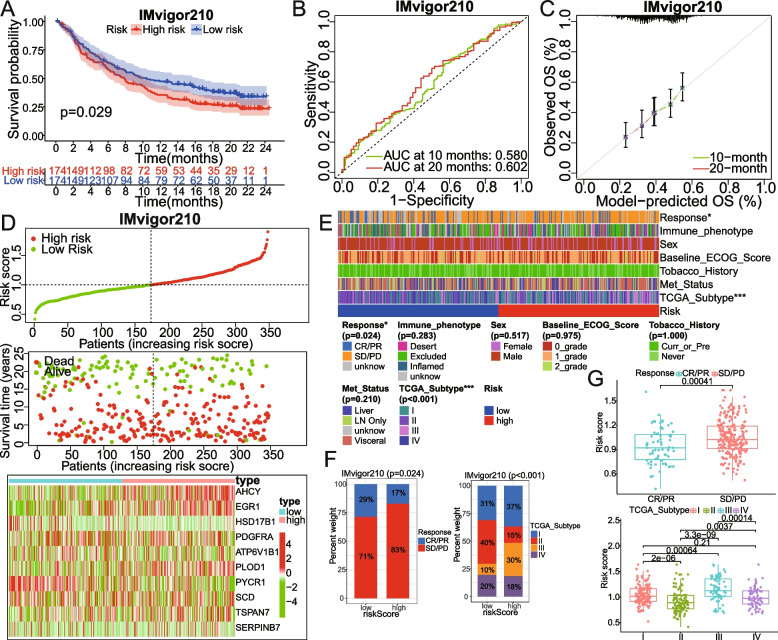
External verification from IMvigor210 cohorts. **A-C** KM, ROC, and calibration curves of our signature were plotted. **D** Top to bottom, the risk score curves, survival status, and model gene expression heatmap are shown. **E–G** The correlation between the risk group and clinical characteristics (including the immunotherapy efficacy, TCGA_subtype, sex, etc.) was carried out by chi-square or Wilcox test. AUC, area under the curve; OS, overall survival; Met_status, metastatic status; Curr_or_Pre, current or previous. *P < 0.05; ***P < 0.001

To evaluate the validity and accuracy of the 10 gene prognostic model in clinical practice, in addition to the GSE13507 and GSE32894 datasets mentioned above, we further conducted a series of analyses for three additional external validation datasets, including GSE48075, GSE31684, and IMvigor210 (Figs. 7 and 8. Based on GSE48075 and GSE31684 cohorts, the findings from the KM, ROC, and calibration curve analysis were highly congruent with the analyses of the model performance described above (Fig. 7A-C, F–H). No correlation was detected between risk groups and several clinical traits (chi-square test, *P* > 0.05; Fig. 7D, I). It could be seen that patients with positive lymph nodes had a higher risk score than those with lymph node metastasis negative via the Wilcox test, which meant that a higher risk score indicated a worse prognosis; whereas risk scores were not significantly different between the presence and absence of FGFR3, P53, RB1, or Ras mutation, distant metastasis, high grade (Fig. 7E, J).

Notably, the IMvigor210 cohort, which is a cohort of 348 MIBC patients treated with Atezolizumab (PD-L1 inhibitor), was included to further evaluate the predictive power of the model in the BCa immunotherapy cohort. For the IMvigor210, the OS of high-risk patients is worse than that of low-risk patients according to the Kaplan–Meier curve (Fig. 8A, *P* < 0.05). Calibration curves for predicting the 10- and 20-month OS showed good performance (Fig. 8B). The predictive performance of OS risk scores was determined using a time-dependent ROC curve with an AUC of 0.580 at 10 months and 0.602 at 20 months (Fig. 8C). We also found that the risk score, survival status, and heatmap of model-gene expression in the validation set were nearly consistent with those in the training set (Fig. 8D). The chi-square test and Wilcox rank test were used to evaluate the correlation of the risk score group with existing clinical variables (Fig. 8E). There was a significant correlation between risk score/groups and treatment response or TCGA_subtype. It is worth mentioning that almost all patients with high risk were in the PD/SD cohort (83%) (Fig. 8F), and patients with PD/SD had higher risk scores than those with CR/PR (Fig. 8G). The analysis results of all these validation datasets also suggested that the 10 gene signature is an important prognostic predictor for BCa patients who received or did not receive immunotherapy.

### The correlation analysis between risk score and immune score, stromal score, expression of immune checkpoint genes, immune-cell infiltration, or the immunotherapy response of BCa patients

**Fig. 9:**
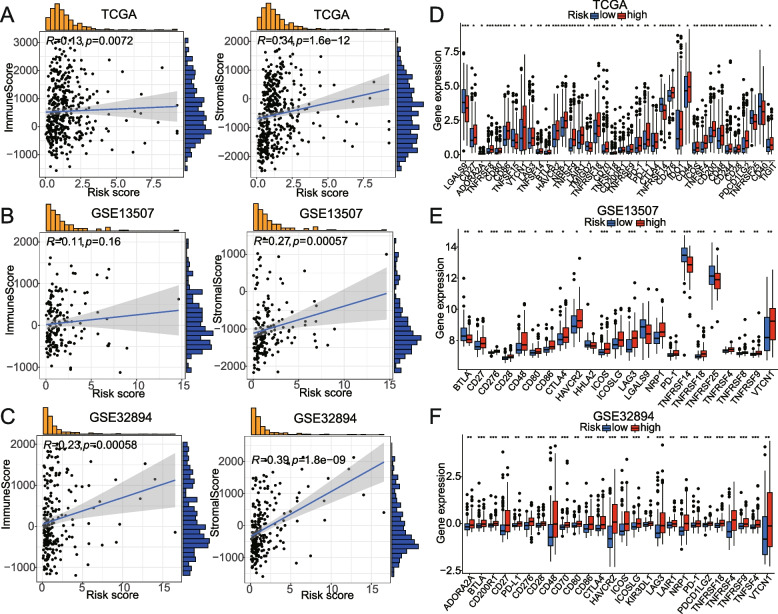
Relationships of the signature with immune score, stromal score (**A-C**) or the expression of immune checkpoint genes (**D-F**) in TCGA, GSE13507, GSE32894 datasets. *P < 0.05; **P < 0.01; ***P < 0.001

Based on TCGA cohorts, we demonstrated that the risk score was positively associated with stromal score (*p* = 1.6e-12), and so is the immune score (*p* = 0.0072) (Fig. 9A). The results are also verified in GSE13507 and GSE32894 datasets (Fig. 9B, C). To further understand better the relationship between the risk score and immune response, we also explored the correlation between risk score and immune checkpoint gene expression. The results showed that in the high-risk group, CD27, CD80, CD86, TNFRSF9, PD-1, and CD48 checkpoint genes were all highly expressed in TCGA, GSE13507, and GSE32894 datasets (Fig. 9D, E, F).

**Fig. 10:**
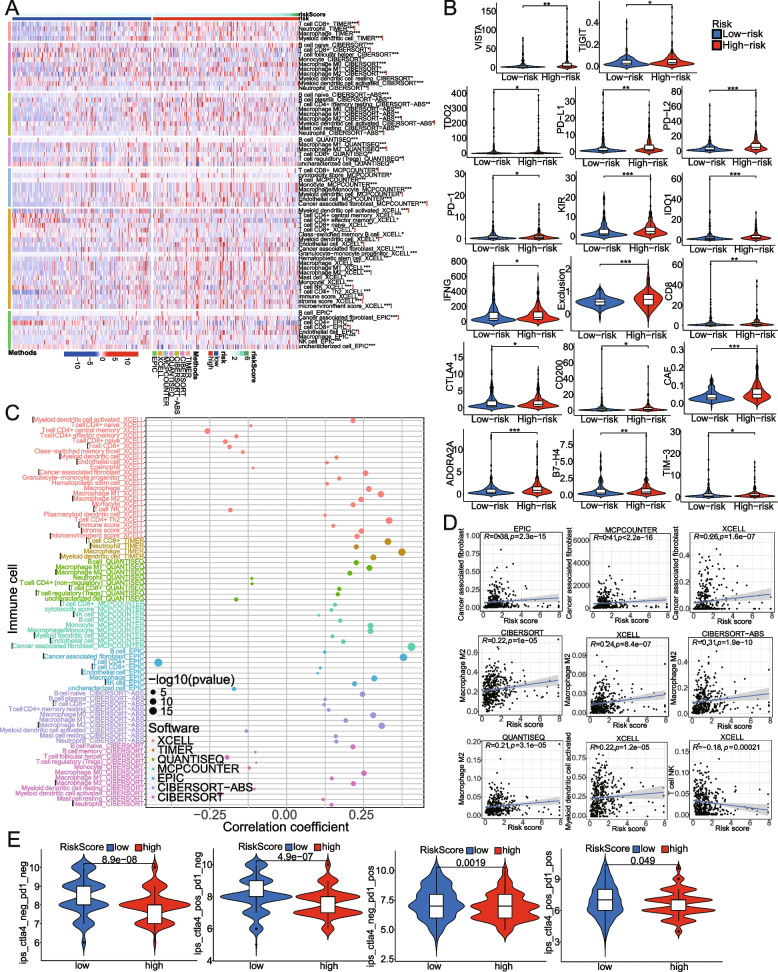
Correlations between the metabolism-associated gene signature and immune-cell infiltration, immune checkpoint immunotherapies. **A** There were significant differences in immune cell infiltration between the high- and low-risk groups. **B** TIDE algorithm was used to predict the immune response of BCa patients to immune checkpoint therapy based on risk score. **C**, **D** Spearman correlation analysis of risk score with infiltrating immune cell abundance was estimated by seven algorithms. **E** We evaluated the predictive value of the model for the anti-PD1/anti-CTLA4 immunotherapy response by the immunophenoscore (IPS). TIDE, the tumor immune dysfunction and exclusion. *P < 0.05, **P < 0.01, ***P < 0.001

The degree of immune cell infiltration in the tumor microenvironment affects tumor occurrence, progression, and therapeutic effect, especially immunotherapy. Based on the 7 immune infiltration algorithms (TIMER, CIBERSORT, CIBERSORT-ABS, QUANTISEQ, MCPCOUNTER, XCELL, and EPIC), we investigated the correlation between risk scores and immune cell infiltration. Heat map of 45 significantly different immune cells in high-risk vs. low-risk groups, including CD8 + T cell, Neutrophil, Myeloid dendritic cell, M2 Macrophage, Tregs, Endothelial cell, Cancer-associated fibroblast, etc., was plotted in Fig. 10A (Wilcoxon test, *P* < 0.01). According to the Tumor Immune Dysfunction and Exclusion (TIDE) [10] tool, the results exhibited that the Exclusion score, CAF score, PD-1, CTLA4, IDO1, and TIGIT score of the high-risk group were higher than those of the low-risk group (Fig. 10B). To further strengthen this result, we applied the IPS method to predict the response of BCa patients to immune checkpoint blockade (PD-1 and CTLA4 therapy). The IPS score of the low-risk group was significantly higher than that of the high-risk group (Fig. 10E). Meanwhile, we performed a correlation analysis between risk scores and immune infiltrating cells using the Spearman test (Fig. 10C, D); this result is consistent with that of the above.

### Screening potential chemotherapeutics drugs

**Fig. 11:**
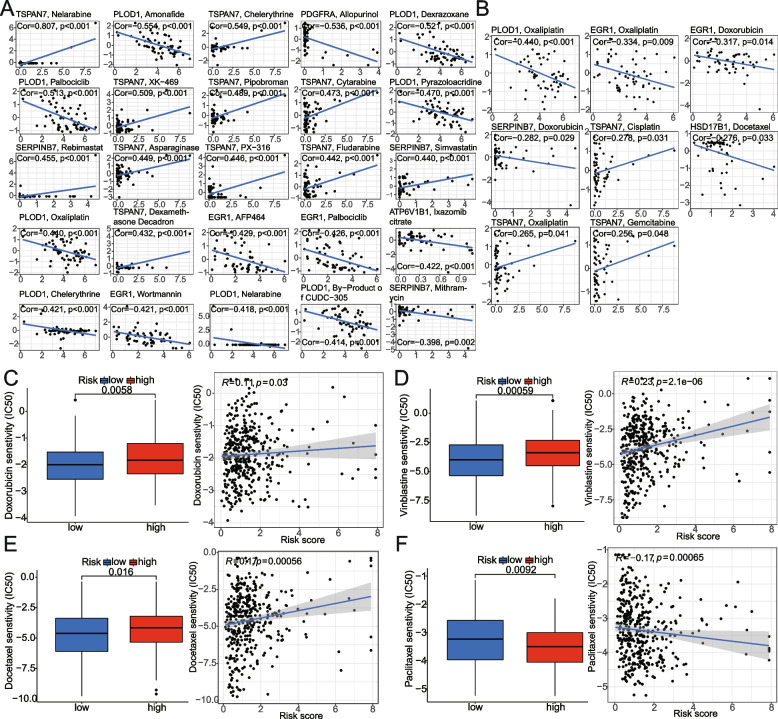
Correlation analysis between the model and chemotherapy agents sensitivity.** A** Scatter plot of relationship between model-genes expression and drug sensitivity IC50. **B** The a significant correlation between commonly used clinical chemotherapeutic drugs IC50 for BCa and the expression of model genes. **C-F** The correlation analysis of risk score with the four commonly used chemotherapy drugs IC50, by the Wilcox and Spearman correlation analyses. Cor, correlation

To study the relationship between these model genes and the sensitivity of chemotherapeutic agents, we determined their expression level and IC50 of the drug across the NCI-60 cell lines. Next, we examined the association of 10 model gene expressions with IC50 for each drug type (Supplementary Table S2) and screened out the top 25 most significant model-gene-related drugs (Fig. 11A). Furthermore, several commonly used clinical drugs (including oxaliplatin, doxorubicin, cisplatin, docetaxel, gemcitabine, and paclitaxel) were analyzed and presented in Fig. 11B. It can be seen that high expression of TSPAN7 has a significant association with the resistance to Nelarabine, Pipobroman, Cytarabine, Asparaginase, Fludarabine, Dexamethasone Decadron, Thiotepa, and Bendamustine (*p* < 0.001); whereas, PDGFRA is sensitive indicators to Aliopurinol (*p* < 0.001). Similarly, the high expression of PYCR1 is significantly associated with Fluorouracil and Seliciclib resistance; on the other hand, its high expression exhibited a sensitivity to the drugs Chelerythrine and Depsipeptide. The high expression of SERPINB7 is a resistant indicator of Rebimastat and Simvastatin; conversely, it is a sensitive marker for Mithramycin and Homoharringtonine. AHCY is the resistance factor of Dabrafenib, Fludarabine, and XL-147. Besides, the high expression of PLOD1 is sensitive index to Dexrazoxane, Palbociclib, Oxaliplatin and Nelarabine; HSD17B1 is sensitive index to Docetaxel and Eribulin mesylate; EGR1 is sensitive to Palbociclib and AFP464; ATP6V1B1 is sensitive to Ixazomib citrate and 3-Bromopyruvate (acid). SCD is sensitive to Panobinostat, while it is insensitive to Salinomycin and SR16157.

Furthermore, through the ‘pRRophetic’ algorithm, we identified that the IC50 of four common chemotherapeutics drugs (Docetaxel, Vinblastine, Paclitaxel, and Doxorubicin) differed between the high- and low-risk groups of the model (*p* < 0.05). The high-risk group compared to the low-risk group had higher IC50 values for Docetaxel, Vinblastine, and Doxorubicin (*P* < 0.05, Wilcox test; Fig. 11C-E); on the contrary, Paclitaxel IC50 values in the high-risk group were higher than low-risk group (Fig. 11F). In addition, we performed a Spearman correlation analysis between the risk scores and IC50 of the above chemotherapy agents. These results verify the above analysis of Wilcox variance (*P* < 0.05; Fig. 11C-F).

### Prediction value of the model for clinical immunotherapeutic efficacy among four BCa immunotherapy cohorts

**Fig. 12:**
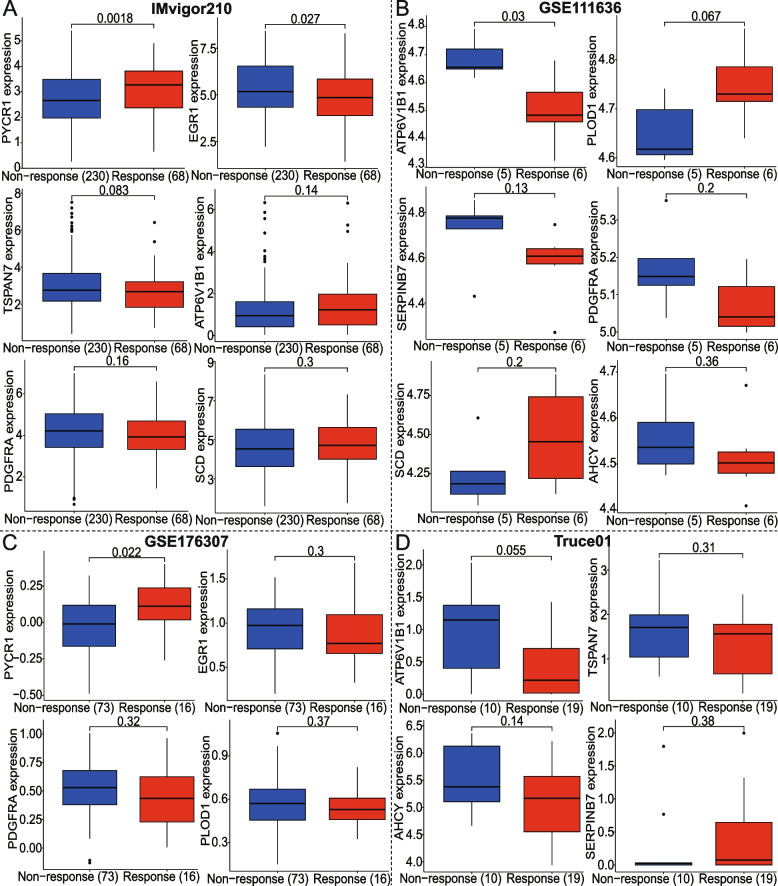
Correlation analysis of each modeled gene expression with immunotherapy response status was performed based on **A** Imvigor210, **B** GSE111636, **C** GSE176307, and **D** our Truce01 datasets

The association analysis between model-gene expression and immunotherapy effectiveness was performed based on four independent real datasets (i.e., Imvigor210, GSE111636, GSE176307, and Truce01) (Fig. 12). We found that the expression levels of PYCR1 were higher and EGR1 was lower in the response group than in non-response across Imvigor210 and GSE176307 datasets. Moreover, TSRAN7 had higher expression levels in BCa patients with non-response according to Imvigor210 and Truce01; and AHCY was highly expressed in the non-response group from GSE111636 and Truce01. Additionally, ATP6V1B1 and SERPINB7 had low expression (*P* = 0.03 and 0.13, respectively); whereas PLOD1 and SCD were high expression (*P* = 0.067 and 0.2, respectively) in the response group from GSE111636 cohort.

**Fig. 13:**
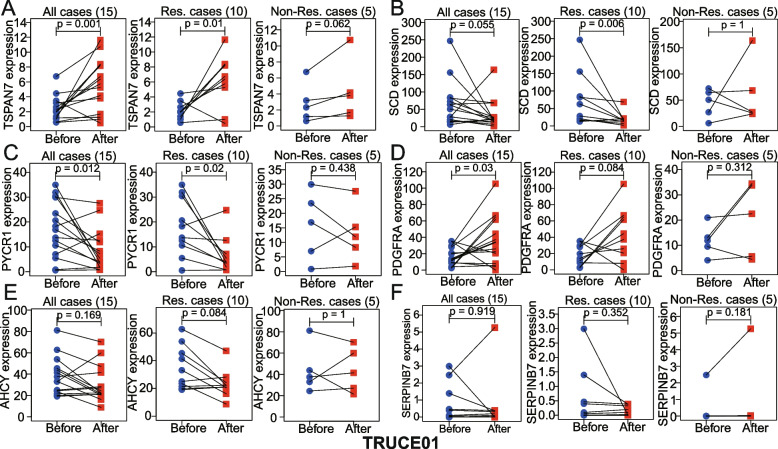
In our Truce01 data, partial model-genes expression, including **A** TSRAN7, **B** SCD, **C** PYCR1, **D** PDGFRA, **E** AHCY, and **F** SERPINB7, was significant changes after immunotherapy in all, response, or non-response cases by paired wilcox test. Res., response; Non-Res., Non-response

In addition, in our ongoing single-arm phase II clinical study (TRUCE01), we also found that in BCa cases that responded to tislelizumab in combination with nab-paclitaxel, expression levels of PYCR1, AHCY, SCD were significantly decreased after treatment, and expression levels of TSPAN7, PDGFRA were significantly elevated after treatment. In non-responsive cases, the expression level of TSRAN7 and SERPINB7 was increased after treatment (Fig. 13). Of the other model genes, no other significant correlations were found (Supplementary Figure S3). Together, these results suggest that the expression of some model genes can help predict immunotherapy response.

### Validation of model-related gene expression by HPA database and qRT-PCR

**Fig. 14:**
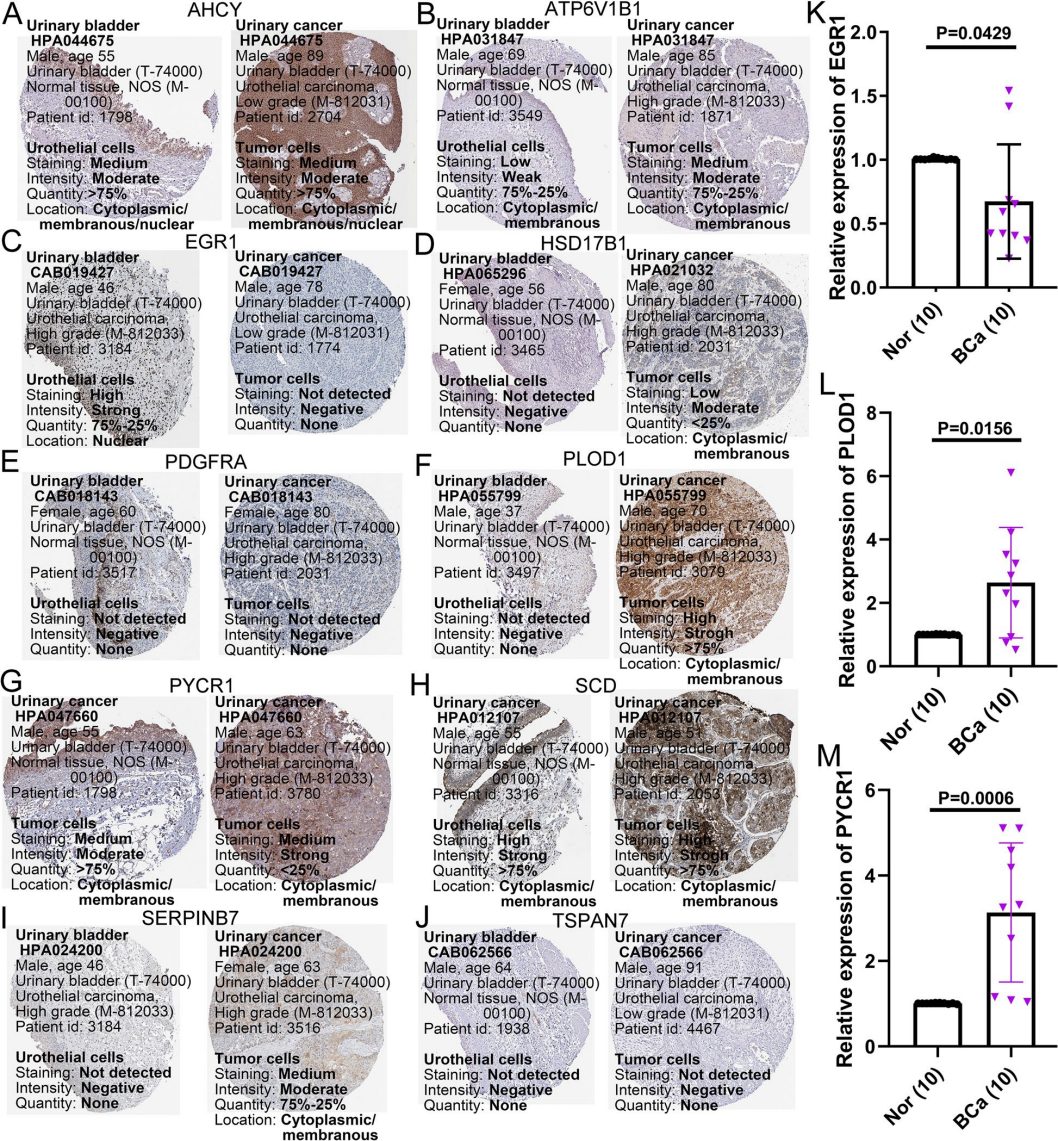
Validation of model gene expression. **A-J** Protein expression of 10 model genes in bladder cancer tissues and normal bladder tissues from the HPA database. **K-M** qRT-PCR was used to detect the mRNA expression of EGR1, PLOD1, and PYCR1 in 10 pairs of BCa and adjacent tissues. BCa, bladder cancer

We then analyzed the protein expression levels of these ten prognostic genes using the Human Protein Atlas (HPA). Compared with normal bladder samples, the protein expression of PLOD1, ATP6V1B1, HSD17B1, SERPINB7, and PYCR1 in bladder cancer tissues was upregulated, while EGR1 in cancer tissues was down-regulated (Fig. 14A-J). Subsequently, mRNA expression levels of EGR1, PLOD1, and PYCR1 were detected in 10 pairs of BCa tissues and corresponding adjacent normal tissues. The qRT-PCR results of these genes were similar to the protein expression results described above (Fig. 14K-M).

## Discussion

In this study, we identified two molecular subtypes from metabolism-related prognostic DEGs through NMF algorithm based on TCGA datasets. And, we found that the two subgroups of patients had different prognosis (C1 was better than C2). Next, we constructed and verified the signature of 10 metabolism-related genes (including PLOD1, SERPINB7, TSPAN7, HSD17B1, PYCR1, PDGFRA, EGR1, AHCY, ATP6V1B1 and SCD) through LASSO, and multivariate Cox regression analyses using TCGA, GSE13507, GSE32894, GSE48075, GSE31684, and Imvigor210 datasets. Furthermore, we systematically investigated and analyzed the correlation of the model with clinical traits, immune infiltration, and sensitivity prediction of chemotherapeutic drugs or immunotherapy. Notely, most genes of the model are closely related to the metabolism of malignant tumors and cancer development.

In January 2021, Wang Z et al. discovered that hypoxia-induced PLOD1 overexpression promotes the malignant phenotype of glioblastoma through NF-κB signaling [15]. In October 2022, Qiongjing Zeng et al. established a prediction model for cervical cancer with five signals including SERPINB7 indicating that SERPINB7 is highly expressed in cervical cancer patients [16]. As early as September 2012, Wuttig D determined that TSPAN7 is a promising prognostic marker for clear cell renal cell carcinoma and indicated that patients with higher expression of the TSPAN7 gene or those with TSPAN7-positive blood vessels in both cores of tissue microarray studies had significantly longer DFS and tumor-specific survival (TSS) [17]. Yu X et al. observed the downregulation of TSPAN7 in BCa tissue samples and cell lines and found that this downregulation was associated with relatively high tumor staging and tumor grade. Western blotting shows that overexpression of TSPAN7 activates Bax, and cleaves caspase-3 and PTEN, but inactivates Bcl-2, p-PI3K, and p-AKT, thereby inhibiting BCa cell growth via the PTEN/PI3K/AKT pathway [18]. HSD17B1 is a steroid-metabolizing enzyme. Previous studies have shown that it is closely related to the occurrence and development of breast and endometrial cancer. PYCR1 is a key enzyme in proline synthesis. In August 2022, Kay EJ et al. discovered that cancer-associated fibroblasts need to synthesize proline by PYCR1 to deposit prototrophic extracellular matrix [19]. Numerous studies have shown that PYCR1 is closely related to the prognosis of various cancers such as bladder cancer, pancreatic ductal adenocarcinoma, renal adenocarcinoma, gastric cancer, prostate cancer, hepatocellular carcinoma, colorectal cancer, etc. Platelet-derived growth subunit A (PDGFA) plays a key role in the development of glioblastoma (GBM). Guo L [20], Gao Z [21], and others also support the high correlation between PDGFRA and the prognosis of patients with bladder cancer. Tao T et al. showed that AHCY showed high potential as a prognostic factor for bladder cancer as a core gene of the co-expression network of lncRNA/mRNA and circRNA/mRNA constructed by WGCNA [22]. The study of Bai S et al. showed that ATP6V1B1 showed excellent prognostic value as one of the nine genes in the hypoxia prognosis model for colorectal cancer.

The BCa samples in the TCGA dataset were then divided into high-risk and low-risk groups based on the median risk score. By analyzing the clustering and risk score, we could find that C2 had a higher risk score and C1 had a lower risk score. K-M survival analysis showed that the OS of the low-risk group was better than the low-risk group. According to the multivariate Cox regression results, the histogram of TNM stage risk score including age and sex was established. The ROC curve was shown to provide favorable predictive performance, and the calibration indicated good agreements between prediction and observation. The nomogram showed that the model was a valid and accurate tool. Our results show that this model can well distinguish BCa patients and predict prognosis, thus helping to formulate the best treatment plan based on risk score. Notably, the signature is well validated in several external BCa datasets (GSE13507, GSE32894, Imvigor210, GSE48075, and GSE31684). To further investigate the underlying mechanisms in the differences, we then performed pathway enrichment analyses for GSEA in each group.

GO analysis showed immunoglobulin receptor binding enrichment in the high-risk group. KEGG pathway enrichment analysis showed that ECM receptor interaction, focal adhesions, actin cytoskeletal regulation, and Leukocytes transendothelial migration were particularly significant enrichment in high-risk groups. Hallmark’s GSEA analysis shows epithelial-mesenchymal transition, angiogenesis, apical junction, myogenesis, hypoxia, inflammatory response, etc. gathering. Although immunoglobulins are about 90% similar at the amino acid level, each subclass has a unique way of antigen binding and immune complex formation. Triggering FcγR-expressing cells leads to a wide range of reactions, includes phagocytosis, antibody-dependent cell-mediated cytotoxicity, and complement activation. Tumor cell-derived IgG may hinder the cytotoxicity of antigen-dependent cells by binding antigens while lacking the ability to activate complement. These findings recommend tumor cell-derived IgG as a potential therapeutic target [23]. CCL2 binds to the homologous receptor CCR2, a signaling pair that has been shown to have a variety of protumorigenic effects, from mediating tumor growth and angiogenesis to recruiting and usurping host stromal cells to support tumor progression [24]. Wei Y et al. elucidate the molecular and cellular basis of KIR3DL3 inhibitory function, demonstrating that the KIR3DL3-HHLA2 pathway is a potential cancer immunotherapy target [25]. However, immunoglobulin binding studies for bladder cancer need to be further studied.ECM plays an important role in tumor shedding, adhesions, degradation, motility, and hyperplasia [26]. Pathology is characterized by abnormal neovascularization and diffuse infiltration of tumor cells. Their role in other cancers has been proven. The expression of ECM is upregulated in prostate cancer tissues [27], and it is involved in the process of tumor invasion and metastasis in gastric cancer [28]. ECM in colorectal cancer promotes epithelial-mesenchymal transformation of cancer cells [29]. Glioblastoma is the deadliest adult brain tumor. The interaction between the ECM and the glioblastoma microenvironment is important in this development [30]. Focal adhesion (FA) is a group of macromolecular proteins that connect specialized actin ends with the extracellular matrix (ECM) and achieve cell migration, which is essential for the process of tumor metastasis [31]. Studies have shown that lncRNA ITGB8-AS1 as ceRNA promotes colorectal cancer growth and migration through integrin-mediated plaque signaling [32]. The hippo component YAP promotes adhesion plaque and tumor aggressiveness by transcriptionally activating THBS1/FAK signaling in breast cancer [33]. Abnormal actin cytoskeletal dynamics have been implicated in a variety of diseases, including cancer [34]. These may be potential targets for future treatments.

Immune cells in the tumor microenvironment (TME) play an important role in tumorigenesis, tumor progression, and metastasis. The tumor microenvironment consists of a heterogeneous population, including the cancer cells themselves, infiltrating immune cells, and stromal cells such as fibroblasts [35]. Correlation analysis showed that risk scores were positively correlated with stromal scores and immune scores. Moreover, the risk scores in the TCGA and several GEO cohorts were positively correlated with PD-L1, POLE2, FEN1, MCM6, MSH6, MSH2, FAP, and TAGLIN.

PD-L1 expression in colorectal cancer tissues was negatively correlated with FOXP3 + cell density, suggesting that PD-L1-expressing cancer cells may affect regulatory T cells in the tumor microenvironment [36]. Based on the results so far, we can define the expression of PD-L1 as a prognostic factor for immunotherapy and a predictor for pembrolizumab [37]. FGFR3 disrupts PD-L1 via NEDD4 to control T cell-mediated immune surveillance of bladder cancer [38]. Overexpression of PD-L1 and PD-1 on tumor cells and tumor-infiltrating lymphocytes, respectively, is associated with poor prognosis in certain human cancers [39]. POLE2 promotes the malignant phenotype of glioblastoma by promoting AURKA-mediated stabilization of FOXM1 [40]. None in bladder cancer. Upregulation and downregulation studies have shown that MCM6 regulates the cell cycle, proliferation, metastasis, immune response, and maintenance of DNA replication systems. MCM6 can also regulate downstream signals, such as MEK/ERK, to promote carcinogenesis [41]. In many cancer cells, MCM6 expression is enhanced and can be used as a therapeutic target. Such as hepatocellular carcinoma [42], breast cancer [43] and gastric cancer [44], and so on. The RR for any cancer was 3.3 (95% CI 2.9 to 3.7) and 2.5 (95% CI 1.7 to 3.2) for path_MSH2 and path_MSH6 carriers, respectively. Older path_MSH2 carriers had a particularly high incidence of urinary tract and prostate cancer. Compared with path_MLH1 and path_MSH2 carriers, we found that path_MSH6 carriers had a lower risk of early-onset cancer and a lower risk of late-onset cancer in addition to an intermediate risk of urinary tract or prostate cancer [45]. MSH6 and bladder cancer (OR, 5.63 [95% CI, 2.75–11.49]) [46]. CircLIFR can interact with MSH2 to positively regulate CDDP sensitivity in bladder cancer through the MutSα/ATM-p73 axis. CircLIFR and MSH2 may be promising therapeutic targets in CDDP-resistant bladder cancer [47]. There is evidence that MSH2 protein levels may contribute to the chemotherapy resistance observed in muscle-invasive bladder cancer. MSH2 has potential as a biomarker to predict response to platinum-based therapy [48]. CAFs express the IL-6 cytokine, and its receptor IL-6R was found in RT4 bladder cancer cells. CM iCAF culture of RT4 bladder cancer cells resulted in significantly enhanced cell growth, migration, and invasion. Importantly, inhibition of CAFs-secreted IL-6 by neutralizing antibodies significantly reversed the IL-6-induced EMT phenotype, suggesting that this cytokine is essential for CAF-induced EMT in human bladder cancer progression. IL-6 expression is upregulated in invasive bladder cancer and correlates with the CAF marker ACTA2 [49]. TAGLN is an antitumor gene in the human bladder. The expression level in normal bladder epithelial cells is higher than that in cancer cells [50].

In addition, immune interactions between tumors and TME play a key role in tumorigenesis and can be used as therapeutic targets for BCa [51]. The composition and abundance of immune cells in TME influence tumor progression and the efficacy of immunotherapy [52]. CD8 + T cells have been recognized as major effector cells of cell-mediated anti-tumor immunity, which kill tumor cells by releasing perforin [53]. Interestingly, we found that CD8 + T cells were significantly higher in the high-risk group. We speculate that this may be related to its dysfunction in a depleted state [54]. A recent study has indicated that the lack of innate inflammatory signaling in tumors leads to the inability to induce transcription factor-regulated functional effector differentiation, further impairing effector function and induced TOX expression and multiple other negative regulators of T cell signaling and function via persistent tumor antigen/TCR stimulation and/or other negative regulatory signals, ultimately leading to dysfunction of tumor-specific CD8 + T cells even before undergoing cell division [55]. Studies have shown that immunosuppressive factors such as Tregs, and cancer-associated fibroblasts evade surveillance and clearance by the immune system through different mechanisms [56]. In peripheral lymphoid organs, Treg cells are classified into resting and effector types, with effector Treg cells secreting IL-10 as an important characteristic [57, 58]. Treg cell physiology is dependent on the expression of GATA3 during inflammation [59]. CD39 and CD73, which are highly expressed on the surface of Treg cells, increase intracellular AMP levels by breaking down ATP into adenosine and binding to adenosine receptor A2A (ADORA2A), activating the CREB pathway, thereby resulting in an anti-inflammatory milieu [60]. Extensive research has been conducted on the role of cancer-associated fibroblasts (CAFs) in solid tumors, particularly in relation to their production of soluble factors such as IL-1α, IL-1β, CXCL1, CXCL12, G-CSF, and IL-6. These factors play a crucial role in recruiting monocytes and myeloid cells, thereby influencing the polarization of immune cells within the tumor microenvironment. Notably, the presence of CAFs has been found to induce the transformation of macrophages into an IL10-mediated tumor-promoting M2 phenotype [61]. Neutrophils recruited to the site of inflammation promote cancer development primarily by increasing DNA damage, angiogenesis, and immunosuppression [62]. Neutrophils can sustain tumor growth through different mechanisms, including inhibiting T cell activation and promoting the proliferation of genetically unstable tumor cells, angiogenesis, and metastasis. These mechanisms include the induction of genetic instability through the production of reactive oxygen species (ROS) and the release of microparticles containing microRNAs miR-23A and miR-155, etc. [63]. Macrophages contribute to tumor progression at different stages, from initiation to distant metastasis formation. Evidence suggests that tumor cells induce macrophages to produce iconic acid and that acidosis in TME promotes immune escape [64]. The research demonstrated by Nixon et al. [65] the immunosuppressive role of pTAMs, which is linked to their capacity to present tumor-associated antigens to CD8 + T cells and induce T cell exhaustion. A recent narrative review [66] proposed that mononuclear macrophages in tumor parenchyma and peritumoral regions of human hepatocellular carcinoma specimens exhibit heightened glycolytic activity, implying that TAMs’ glucose uptake facilitates tumor advancement. Several metabolites produced in glucose and lipid metabolism pathways, as well as those derived from amino acids, can also function as signaling molecules, promoting scavenging and anti-inflammatory functions in tumor-associated macrophages (TAMs). The pro-tumor activities exhibited by TAMs hinder patient responses to conventional chemotherapy, radiotherapy, and immunotherapy. Cancer cells can promote the formation and survival of endothelial cell tubes, at least in part through the PI3KAkt signaling pathway, thereby altering the microenvironment in favor of tumor growth [67]. This is consistent with our findings of abundant CD8 + T cells, Neutrophils, Myeloid dendritic cells, M2 macrophages, Tregs, Endothelial cells, and Cancer-associated fibroblasts in patients in the BCa high-risk group. Therefore, we speculate that metabolic prognostic models may influence survival outcomes for BCa by altering ECM and immunosuppressive cells.

Despite this work were also similar to the previous study [68, 69], thereby lacking a certain amount of innovation, the model built by us was more streamlined and validated by multiple datasets, and its association analyses from multiple angles were performed. Although, a series of integrative analysis of multiple datasets from open databases (i.e., TCGA, GEO, TIDE, CellMiner, and TIMER) and our mRNA sequencing data (TRUCE01), as well as validation by immunohistochemistry and qRT-PCR were carried out, the main limitation of the research is that it lacks some functional experiments in vivo and in vitro to clarify the relevant molecular mechanisms of these modeled genes. Furthermore, further prospective studies are required to validate the clinical value of this metabolism-based molecular subtype and its signature.

Notably, our study showed unique immune landscapes, immune checkpoint gene expression, and immunotherapy responses between the high-risk and low-risk groups. In addition, we also calculated IC50 values to explore the chemotherapy drug’s sensitivity for BCa and screened candidate small molecule compounds. Furthermore, the TIDE and IPS algorithms were all used to predict the immunotherapy response of our model. Compared with the low-risk group, the high-risk BCa patients may be non-responsive and advanced for the immunotherapy. Additionally, the research also found that these model genes may act as a promising biomarker for predicting the efficacy of immunotherapy in BCa patients based on four independent real immunotherapy datasets, including IMvigor210, GSE111636, GSE176307, and our Truce01. These findings may provide suitable treatment options for patients with BCa. The proteins and mRNA expression of EGR1, PLOD1, and PYCR1 were also detected by the HPA database and qRT-PCR.

## Conclusions

In summary, we developed and validated a new signature based on metabolism-related genes that may serve as a predictor for BCa prognosis, chemotherapy, or immunotherapy sensibility. Therefore, there are direct implications for guiding clinical oncology practice.

### Supplementary Information

**Additional file 1: Supplementary Figure S1.** To achieve a preferable clustering performance, we set the k cluster range from 2 to 10 using the “NMF” R package. **Supplementary Figure S2.** Kaplan–Meier analyses of OS in different clinicopathological subgroups stratified by (A) age, (B) gender, (C) stage, and (D) stage_T. **Supplementary Figure S3.** Based on (A) Imvigor210, (B) GSE111636, (C) GSE176307, and (D) our Truce01 cohorts, the partial genes expression of the model were not statistically different between response and non-response group. (E-H) The four model genes (including HSD17B1, EGR1, PLOD1, and ATP6V1B1) showed no difference in expression before and after immunotherapy combined with nab-paclitaxel. Res., Response; Non-Res., Non-Response.

**Additional file 2: Supplementary Table S1.** Based on (A) TCGA, (B) GSE13507, and (C) GSE32894 datasets, GO, KEGG, or HALLMARK enrichment analysis were compared between the high-risk and low-risk cohorts using the GSEA. 

**Additional file 3: Supplementary Table S2. **Drug sensitivity analysis of metabolism-associated model genes based on the CellMiner database. 

## Data Availability

The original contributions presented in the study are included in the article/Supplementary Material. Further inquiries can be directed to the corresponding authors. This study generated sequencing data that is available at TCGA (https://portal.gdc.cancer.gov) or NCBI GEO (https://www.ncbi.nlm.nih.gov/geo/).

## Abbreviations

BCa: Bladder cancer
TCGA: The cancer genome atlas
GEO: Gene expression omnibus
GSEA: Gene set enrichment analysis
GO: Gene ontology
KEGG: Kyoto encyclopedia of genes and genomes
ROC: Receiver operating characteristic
ICI: Immune checkpoint inhibitor
PD-L1: Programmed cell death-ligand 1
PD-1: Programme death 1
OS: Overall survival curves
PFS: Progression free survival
DFS: Disease free survival
DSS: Disease specific survival
LASSO: Least absolute shrinkage and selection operator
AUC: Area under the ROC curve
DCA: Decision curve analysis
TIDE: The tumor immune dysfunction and exclusion
TIS: The tumor inflammation signature
MF: Molecular function
CC: Cellular component
BP: Biological process
DEGs: Differentially expressed genes
IPS: Immunophenoscore
IC50: Semi-maximum inhibitory concentrations
