# Supplementary material for: Construction and validation of a metabolism-associated gene signature for predicting the prognosis, immune landscape, and drug sensitivity in bladder cancer
Source: BMC Med Genomics. 2023 Oct 26;16:264. doi: 10.1186/s12920-023-01678-6 (PMC10601123; doi:10.1186/s12920-023-01678-6)
Supplement: Supplementary file 1 — Additional file 1: Supplementary Figure S1. To achieve a preferable clustering performance, we set the k cluster range from 2 to 10 using the “NMF” R package. Supplementary Figure S2. Kaplan–Meier analyses of OS in different clinicopathological subgroups stratified by (A) age, (B) gender, (C) stage, and (D) stage_T. Supplementary Figure S3. Based on (A) Imvigor210, (B) GSE111636, (C) GSE176307, and (D) our Truce01 cohorts, the partial genes expression of the model were not statistically different between response and non-response group. (E-H) The four model genes (including HSD17B1, EGR1, PLOD1, and ATP6V1B1) showed no difference in expression before and after immunotherapy combined with nab-paclitaxel. Res., Response; Non-Res., Non-Response. [file 12920_2023_1678_MOESM1_ESM.docx]

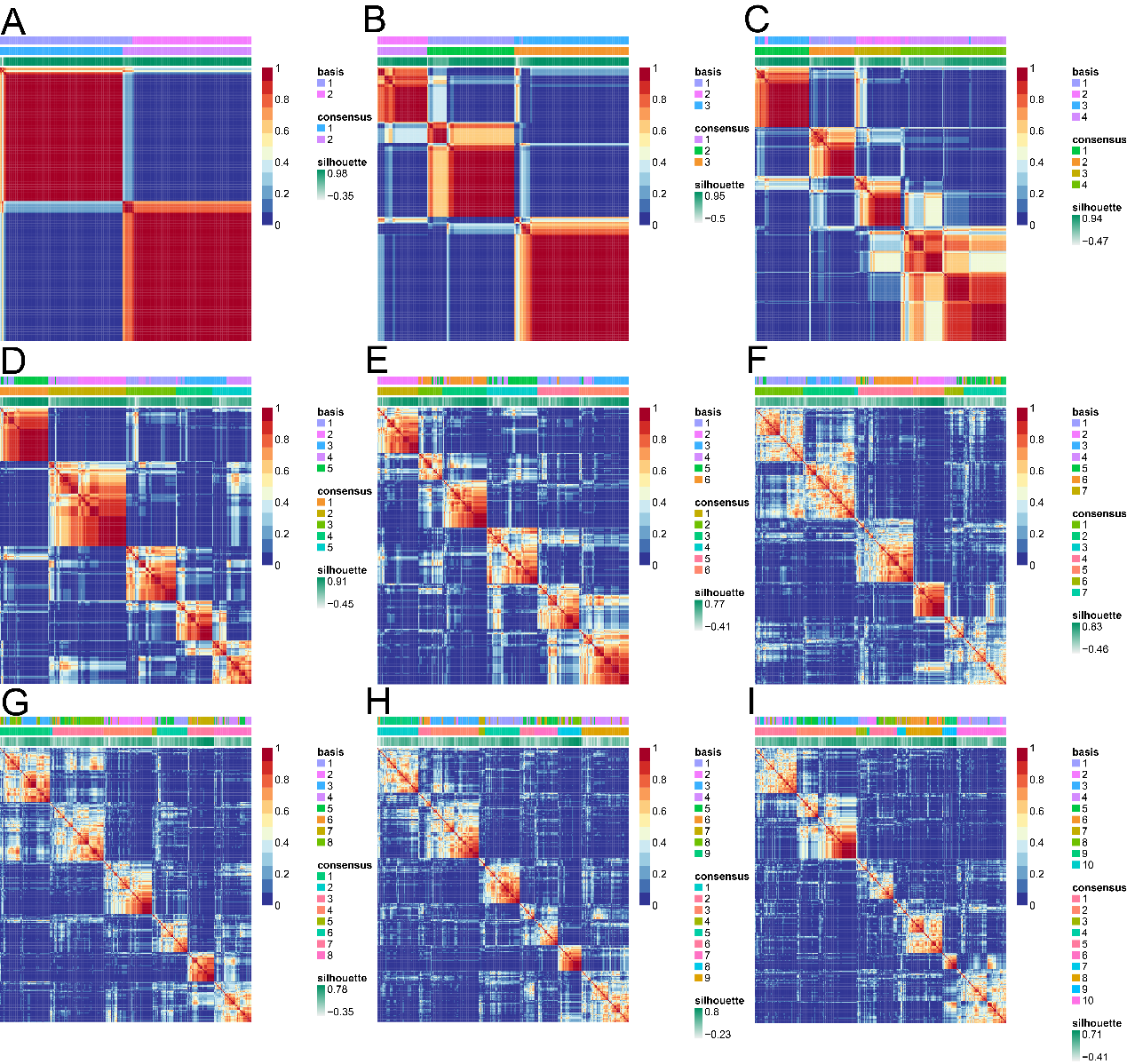


**Supplementary Figure S1.** To achieve a preferable clustering performance, we set the k cluster range from 2 to 10 using the “NMF” R package.


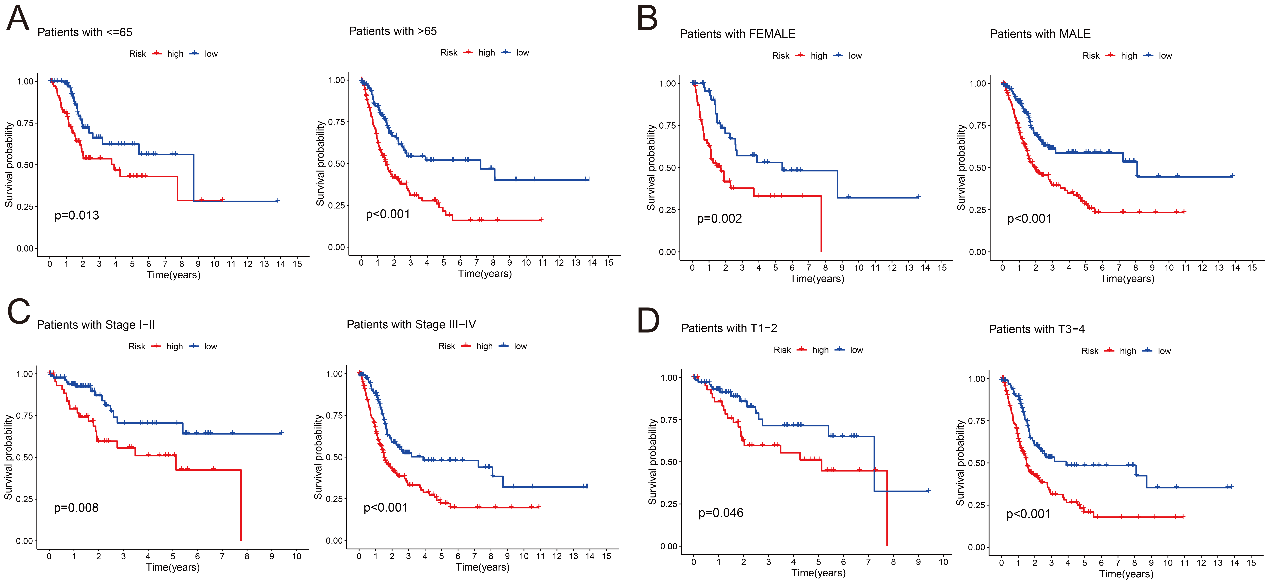


**Supplementary Figure S2**. Kaplan–Meier analyses of OS in different clinicopathological subgroups stratified by **(A)** age, **(B)** gender, **(C)** stage, and **(D)** stage_T.


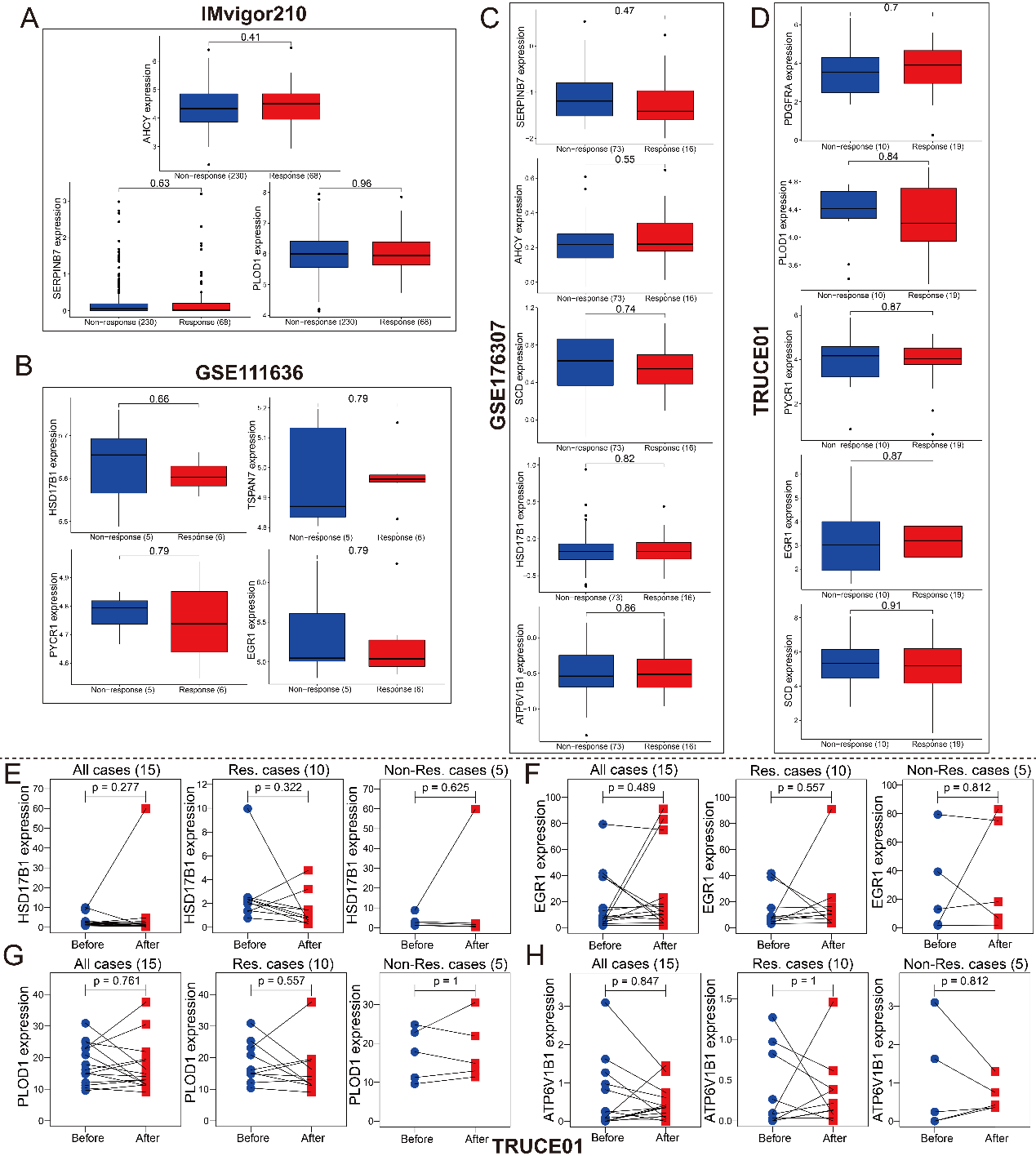


**Supplementary Figure S3.** Based on **(A)** Imvigor210, **(B)** GSE111636, **(C)** GSE176307, and **(D)** our Truce01 cohorts, the partial genes expression of the model were not statistically different between response and non-response group. **(E-H)** The four model genes (including HSD17B1, EGR1, PLOD1, and ATP6V1B1) showed no difference in expression before and after immunotherapy combined with nab-paclitaxel. Res., Response; Non-Res., Non-Response.
